# Swallowing Outcomes Following Voice Therapy in Multiple System Atrophy with Dysphagia: Comparison of Treatment Efficacy with Parkinson’s Disease

**DOI:** 10.1007/s00455-021-10265-9

**Published:** 2021-03-05

**Authors:** Alyssa Park, Su-Jeong Jang, No-Eul Kim, Tae-Hui Kim, Young Ho Sohn, HyangHee Kim, Sung-Rae Cho

**Affiliations:** 1 Graduate Program in Speech and Language Pathology, Yonsei University College of Medicine, Seoul, 03722 Korea; 2 Department and Research Institute of Rehabilitation Medicine, Yonsei University College of Medicine, Seoul, 03722 Korea; 3 Department of Neurology, Yonsei University College of Medicine, Seoul, 03711 Korea; 4 Brain Korea 21 PLUS Project for Medical Science, Yonsei University College of Medicine, Seoul, 03722 Korea; 5 Graduate Program of NanoScience and Technology, Yonsei University College of Medicine, Seoul, 03722 Korea; 6 Rehabilitation Institute of Neuromuscular Disease, Yonsei University College of Medicine, Seoul, 03722 Korea

**Keywords:** Deglutition, Deglutition disorders, Lee silverman voice treatment, Parkinsonism, Multiple system atrophy

## Abstract

Difficulties with speech and swallowing occur in patients with Parkinsonism. Lee Silverman Voice Treatment (LSVT) is proven as an effective treatment for speech and swallowing function in idiopathic Parkinson’s disease (IPD). The effect of LSVT on swallowing function in multiple system atrophy-cerebellar type (MSA-C) is unknown. We sought to determine LSVT’s effect on swallowing function in MSA-C patients compared to IPD patients. LSVT-LOUD was performed on 13 patients with Parkinsonism (6 IPD and 7 MSA-C). Maximum phonation time (MPT), voice intensity, Speech Handicap Index-15 (SHI-15), Swallowing-Quality of Life (SWAL-QOL), National Institutes of Health-swallowing safety scale (NIH-SSS), and videofluoroscopic dysphagia scale (VDS) before and after LSVT were analyzed and reevaluated three months after treatment. The IPD and MSA-C groups showed significant improvements in overall speech and swallowing measures after LSVT. In particular, pharyngeal phase score and total score of VDS improved significantly in both groups. A two-way repeated-measure ANOVA revealed a significant main effect for time in the MPT, voice intensity, NIH-SSS, pharyngeal phase score and total score of VDS, psychosocial subdomain of SHI-15, and SWAL-QOL. The MSA-C group experienced less overall improvement in swallowing function, but the two groups had an analogous pattern of improvement. In conclusion, LSVT is effective for enhancing swallowing function, particularly in the pharyngeal phase, in both IPD and MSA-C patients. This study demonstrated that LSVT elicits significant improvements in MSA-C patients. We deemed LSVT to be an effective treatment for IPD and MSA-C patients who suffer from dysphagia.

**Supplementary Information:**

The online version contains supplementary material available at 10.1007/s00455-021-10265-9.

## Introduction

Swallowing problems frequently arise with the progression of idiopathic Parkinson’s disease (IPD), causing poor quality of life [1, 2]. In the oral phase, problems with tongue movement hinder the anterior–posterior movement of food, and difficulty in closing the lips causes drooling. In the pharyngeal phase, issues such as pharyngeal rigidity, delayed swallowing reflex, and impaired laryngeal elevation occur [3]. Further, weakness of pharyngeal movement can cause food residue to accumulate in the pharynx [4]. Patients with multiple system atrophy (MSA) have laryngeal dysfunction, causing laryngeal stridor [5]. Laryngeal stridor is caused by the inability to adduct the vocal cords, mainly due to atrophy of the posterior cricoarytenoid muscles of the larynx [6]. In addition, patients with MSA have pharyngeal dysfunction, including impaired tongue base to pharyngeal wall contact, swallow initiation, pharyngeal pooling, laryngeal closure and laryngeal elevation, penetration, aspiration, and food residue in the valleculae and/or the pyriform sinus after the swallow [7].

Various efforts in the areas of healthcare and rehabilitation have been made to address these issues. Among them, voice therapy using Lee Silverman Voice Treatment (LSVT) has been applied to patients with IPD. The LSVT-LOUD program is based on the hypothesis that intensive phonation exercises can improve the exercise capacity of respiratory and laryngeal muscles, thus, ameliorating decreased voice intensity and monotonous speech of patients with dysarthria [8]. Studies of LSVT-LOUD in practice have demonstrated that the effects of respiration and vocalization are associated with increased voice strength, improved sound quality, and increased maximum phonation time (MPT) [9, 10], eliciting improvement in swallowing function in IPD [11, 12]. Specifically, the pilot study on participants with PD [11] demonstrated significant improvements in pharyngeal phase parameters, including increased opening duration and maximal opening of the pharyngoesophageal sphincter during ingestion of a 20 ml fluid bolus, reduced residue during ingestion of a thick bolus, and decreased pharyngeal area at rest post-LSVT-LOUD treatment.

However, it is unknown whether LSVT-LOUD influences swallowing in MSA, which shares many symptoms with IPD, and whether these effects are maintained after termination of treatment for a long-term period of time. Therefore, we examined the difference in improvements of speech and swallowing function through LSVT-LOUD in IPD and MSA cerebellar type (MSA-C) patients. The study’s hypotheses were that the IPD and MSA-C groups will demonstrate improved pharyngeal phase post-LSVT-LOUD treatment, and that they will show sustained a treatment effect on swallowing functions at follow-up evaluation. We also sought to determine LSVT’s effect on swallowing function in MSA-C patients compared to IPD patients.

## Materials and Methods

### Participants

A total of 13 patients with Parkinsonism (6 IPD and 7 MSA-C) were recruited with informed written consent. The inclusion criteria were (1) diagnosis of IPD or MSA-C by a neurologist or physiatrist and (2) complaint of difficulty in swallowing. All subjects were taking antiparkinson medication, and the medication was not changed during the intervention. Exclusion criteria were (1) suspected secondary Parkinsonism due to drugs or infection, (2) other neurological damage or disease that caused a swallowing disorder, and (3) additional swallowing intervention in the duration of the experiment.

Mean age of participants is 66.69 ± 6.58 years. The mean age of the IPD group was 7.00 ± 7.12 years and that of the MSA-C group was 63.86 ± 4.91 years. The mean stages of Hoehn and Yahr scale for both patient groups, IPD, and MSA-C were 3.77 ± 1.64, 3.00 ± 1.54, and 4.43 ± 1.51, respectively. The mean post-onset times for both patient groups, IPD, and MSA-C were 5.92 ± 7.15 years, 1.00 ± 8.92 years, and 2.43 ± 2.29 years, respectively. For all patients, IPD, and MSA-C, the mean scores of the Mini-Mental State Examination were, respectively, 25.62 ± 4.77, 27.00 ± 4.05, and 24.43 ± 5.31. All participants had been prescribed oral diets with no modifications and had not previously been treated with gastrostomy tube feeding. Speech and swallowing parameters were reevaluated in 13 patients (6 IPD and 7 MSA-C) with a follow-up assessment after approximately three months. This study was approved by the Institutional Review Board (NO. 4-2012-0483).

### Treatment

LSVT-LOUD^®^, a speech-behavior therapy, aims to increase vocal cord adduction and voice intensity. A standardized program was conducted by a qualified speech-language pathologist. A total of 16 sessions were held for four consecutive days per week for one month. During the treatment period, the patient was instructed to practice for 5–10 min as homework and 10–15 min on untreated days.

### Data Collections and Analysis

Subjects who agreed to participate in the study completed a questionnaire about quality of life and then received speech and swallowing evaluations. After the pre-evaluation, speech therapy was performed. After the intervention, post-evaluation was carried out in the same manner as the pre-evaluation. Approximately three months after the post-evaluation, follow-up evaluation was carried out in the same manner as the pre-evaluation.

### Voice Data Collection

Sound data were recorded in a quiet room with an ambient noise of less than 50 dB. A 10-cm distance was maintained between the mouth and the condenser microphone (SONY ECM-MS907, SONY Corp., Tokyo, Japan). The program was digitized at a sampling rate of 44.1 Hz and 16-bit quantization. The recording level was fixed at -12 dB. Data were analyzed using Praat, a motor speech protocol that is a module of the Computerized Speech Lab model 5105. MPT and vocal intensity were collected [13, 14]. For the MPT measurement, the subject was asked to produce the long vowel /a/. This vocalization was measured three times, and the longest MPT was recorded. To determine voice intensity, we measured the sentence containing the functional word “Hello” (*annyeonghaseyo*). Voice data for each patient was collected by the same speech-language pathologist for pre-treatment, post-treatment, and follow-up assessment. Pre-treatment assessment was conducted between one and seven days prior to starting treatment, post-treatment assessment was conducted between one and seven days after finishing treatment, and follow-up assessment was conducted approximately three months after post-treatment assessment.

### Data Collection of Quality of Life Related to Speech and Swallowing

To assess the effects of patient speech on daily life, a short form of the Speech Handicap Index-15 (SHI-15) was used as a self-report assessment tool. SHI-15 consists of two subcategories and 15 questions. Higher scores indicate a lower quality of life associated with speech [15]. Swallowing-related quality of life (SWAL-QOL) was measured to assess the quality of life of patients associated with oropharyngeal swallowing disorders [16, 17]. SWAL-QOL consists of 11 subcategories and 44 questions. Higher scores indicate a higher quality of life associated with swallowing impairment [17].

### Swallowing Data Collection

During the VFSS, subjects were given 15 ml of a thin liquid solution mixed with barium sulfate and swallowed according to the instructions of the tester. Previous studies have shown that patients with PD, due to the rigidity of the pharyngeal muscles, have delayed elevation of the hyoid bone and delayed onset of airway closure when swallowing a large volume of liquid [18]. Additionally, studies have shown that patients with oropharyngeal dysphagia exhibit indicators of unsafe swallowing most commonly with liquid boluses, and that boluses with thicker viscosities promote swallowing safety [19]. As the patients in this study did not have high-severity dysphagia, based on previous studies, this study chose to analyze the more sensitive thin liquid bolus swallows. The test was assessed using the National Institutes of Health-swallowing safety scale (NIH-SSS) and Videofluoroscopic Dysphagia Scale (VDS). NIH-SSS assesses the stability of swallowing, which is recorded and quantified by evaluating residue, penetration, and aspiration [20]. A higher score on the scale of 0 to 8 indicates more severe dysphagia.

VDS quantifies the swallowing function of the oropharyngeal stage, which allowed us to examine the effects of treatment on swallowing function conveniently and closely. A total of 14 items were classified into oral phase (7 items) and pharyngeal phase (7 items). The total score ranged from 0 to 100 (oral phase 40, pharyngeal phase 60), with a higher score indicating more severe dysphagia [21].

### Statistical Analysis

Statistical analysis was performed using the IBM SPSS (Statistical Package for the Social Science, version 25.0) for Windows program. A repeated-measure ANOVA and a two-way repeated-measure ANOVA were conducted to determine whether there were any differences in speech (MPT and voice intensity), swallowing function (NIH-SSS and VDS), and self-reports (SHI-15 and SWAL-QOL) before and after LSVT-LOUD treatment in patients with IPD and MSA-C.

The scores of NIH-SSS and VDS were measured by three speech-language pathologists. Pearson correlation analysis was performed to obtain the inter-rater reliability between the evaluators. To assess the reliability of the evaluators, 20% of the data were randomly selected, and correlations were measured using the Pearson correlation analysis. The inter-rater reliability and intra-rater reliability of total NIH-SSS and VDS were assessed using Intra-Class Correlation per Morton and Dobson (see Supplementary Results).

## Results

### Comparison of Speech and Swallowing Function in IPD and MSA-C Groups Before and After LSVT

A two-way repeated-measure ANOVA revealed a significant main effect for time for the following scores: MPT (*F*(2, 22) = 15.622, *p* < 0.001), voice intensity (*F*(2, 22) = 7.515, *p* = 0.003), NIH-SSS (*F*(2, 22) = 3.568, *p* = 0.046), and VDS’s pharyngeal phase (*F*(2, 22) = 12.900, *p* < 0.001) and total score (*F*(2, 22) = 7.703, *p* = 0.003) as shown in Table 1, as well as laryngeal elevation (*F*(2, 22) = 7.391, *p* = 0.004), pharyngeal transit time (*F*(2, 22) = 6.320, *p* = 0.007), and coating on the pharyngeal wall (*F*(2, 22) = 4.410, *p* = 0.025) as shown in Table 2. A two-way repeated-measure ANOVA also revealed a significant group effect for MPT (*F*(1, 11) = 8.632, p = 0.013) as shown in Table 1 and a significant time × group interaction for premature bolus loss of VDS (*F*(2, 22) = 6.953, p = 0.005) as shown in Table 2.

**Table 1:** Comparison of speech and swallowing function in IPD and MSA-C groups before and after LSVT

Variables	IPD			MSA-C			Time		Group		Time*Group	
	Pretest	Posttest	Follow-up	Pretest	Posttest	Follow-up	*F*	*p*	*F*	*p*	*F*	*p*
Speech												
MPT	13.01 ± 5.85	20.89 ± 9.05**	18.88 ± 7.86*	7.09 ± 3.31	10.36 ± 2.50*	10.32 ± 1.96**	15.622	.000***	8.632	.013*	2.380	.116
Voice intensity	75.68 ± 4.80	78.70 ± 2.35	78.93 ± 2.84	72.36 ± 6.73	78.24 ± 1.59*	76.42 ± 3.77*	7.515	.003*	1.360	.268	0.726	.495
Swallowing												
NIH-SSS	1.83 ± 1.83	0.67 ± 0.51	0.83 ± 0.75	1.71 ± 0.95	1.29 ± 0.48	1.14 ± 1.06	3.568	.046*	0.430	.526	0.586	.565
VDS												
Oral phase	5.58 ± 12.95	0.75 ± 1.25	1.75 ± 1.75	4.28 ± 6.89	0.64 ± 1.18	5.42 ± 7.21	1.500	.245	0.106	.751	0.540	.590
Pharyngeal phase	25.58 ± 14.76	8.08 ± 4.34*	9.50 ± 6.45*	24.78 ± 12.08	14.57 ± 7.66*	17.50 ± 8.18	12.900	.000***	1.235	.290	1.285	.297
Total score	31.16 ± 26.48	8.83 ± 4.79	11.25 ± 8.09	29.07 ± 16.32	15.21 ± 7.66*	22.92 ± 14.03	7.703	.003**	0.781	.396	1.067	.361

Data are presented as mean ± SD

*LSVT* Lee Silverman Voice Treatment, *IPD* Idiopathic Parkinson’s disease, *MSA-C* Multiple system atrophy-Cerebellar type, *MPT* Maximum phonation time, *NIH-SSS* National Institutes of Health-swallowing safety scale, *VDS* Videofluoroscopic Dysphagia Scale

*P < 0.05, **P < 0.01, ***P < 0.001

^*^significant results were returned for pretest and posttest, Pretest Follow-up

**Table 2:** Comparison of sub-item scores of VDS in IPD and MSA-C groups before and after LSVT

VDS	IPD			MSA-C			Time		Group		Time*Group	
	Pretest	Posttest	Follow-up	Pretest	Posttest	Follow-up	*F*	*p*	*F*	*p*	*F*	*p*
Lip closure	0.33 ± 0.81	0 ± 0	0 ± 0	0 ± 0	0 ± 0	0.57 ± 1.51	0.515	.604	0.118	.738	1.309	.290
Bolus formation	1.00 ± 2.44	0 ± 0	0 ± 0	0.43 ± 1.13	0 ± 0	0.85 ± 2.26	0.775	.473	0.046	.835	0.775	.473
Mastication	1.33 ± 3.26	0 ± 0	0 ± 0	0.57 ± 1.51	0 ± 0	1.71 ± 2.13	1.375	.274	0.286	.604	2.010	.158
Apraxia	0 ± 0	0 ± 0	0 ± 0	0 ± 0	0 ± 0	0 ± 0	N/A	N/A	N/A	N/A	N/A	N/A
Tongue to palate contact	1.67 ± 4.08	0 ± 0	0 ± 0	0.71 ± 1.89	0 ± 0	1.42 ± 2.43	1.037	.371	0.064	.805	1.037	.371
Premature bolus loss	0.75 ± 1.25	0.75 ± 1.25	1.75 ± 1.75	2.14 ± 1.90	0.64 ± 1.18*	0.85 ± 1.18	3.271	.057	0.033	.860	6.953	.005**
Oral transit time	0.50 ± 1.22	0 ± 0	0 ± 0	0.43 ± 1.13	0 ± 0	0 ± 0	2.014	.157	0.012	.915	0.012	.988
Triggering of Pharyngeal swallow	3.75 ± 1.83	3.75 ± 1.83	3.75 ± 1.83	3.85 ± 1.70	3.85 ± 1.70	3.85 ± 1.70	N/A	N/A	0.012	.915	N/A	N/A
Vallecular residue	1.33 ± 1.63	1.33 ± 1.03	1.00 ± 1.09	1.43 ± 0.97	0.86 ± 1.06	0.57 ± 0.97	0.961	.398	0.464	.510	0.272	.764
Laryngeal elevation	7.50 ± 3.67	1.50 ± 3.67*	3.00 ± 4.64	7.71 ± 3.40	5.14 ± 4.81	6.42 ± 4.39	7.391	.004**	1.641	.227	1.426	.262
Pyriform sinus residue	3.00 ± 3.67	0 ± 0	0.75 ± 1.83	1.92 ± 3.54	1.28 ± 2.19	0.64 ± 1.70	3.055	.067	0.001	.972	0.999	.385
Coating on the pharyngeal wall	4.50 ± 4.93	1.50 ± 3.67	0 ± 0	1.29 ± 3.40	0 ± 0	0 ± 0	4.410	.025*	2.327	.155	1.265	.302
Pharyngeal transit time	1.50 ± 2.51	0 ± 0	0 ± 0	2.57 ± 3.20	0 ± 0	0 ± 0	6.320	.007**	0.438	.522	0.438	.522
Aspiration	4.00 ± 4.89	0 ± 0	1.00 ± 2.44	6.00 ± 4.89	3.43 ± 4.72	6.00 ± 4.89	3.357	.053	3.761	.079	0.698	.508

Data are presented as mean ± SD

*LSVT* Lee Silverman Voice Treatment, *IPD* Idiopathic Parkinson’s disease, *MSA-C* Multiple system atrophy-Cerebellar type, *VDS* Videofluoroscopic Dysphagia Scale

*P < 0.05, **P < 0.01

^*^significant results were returned for pretest and posttest, Pretest Follow-up

### Changes in Speech and Swallowing Function in IPD and MSA-C Groups After LSVT

In the IPD group, a repeated-measure ANOVA revealed a significant difference between pretest and posttest and between pretest and follow-up for MPT (*p* = 0.005;* p* = *0.0*41). Regarding swallowing function, there were significant differences found between pretest and posttest and between pretest and follow-up for VDS’s pharyngeal phase (*p* = 0.049; *p* = *0.0*16). VDS showed significant differences between pretest and posttest for laryngeal elevation (*p* = 0.025) and between pretest and follow-up for premature bolus loss (*p* = *0.0*25) as shown in Tables 1 and 2.

Meanwhile, in the MSA-C group, significant differences were revealed between pretest and posttest and between pretest and follow-up for MPT (*p* = 0.023; *p* = 0.006) and voice intensity (*p* = 0.039; *p* = 0.041). Regarding swallowing function, there were significant differences between pretest and posttest for VDS’s pharyngeal phase (*p* = 0.030), total score (*p* = 0.043), and premature bolus loss (*p* = 0.038). However, no significant differences were revealed between pretest and follow-up for the MSA-C group’s swallowing functions as shown in Tables 1 and 2

### Comparison of Speech and Swallowing-related Quality of Life in IPD and MSA-C Groups Before and After LSVT

A two-way repeated-measure ANOVA revealed a significant main effect for time for the following variables: SHI-15′s psychosocial score (*F*(2, 22) = 4.156, *p* = 0.029), and SWAL-QOL’s symptom frequency (*F*(2, 22) = 8.179, *p* = 0.002), eating duration (*F*(2, 22) = 7.431, *p* = 0.003), and total score (*F*(2, 22) = 6.900, *p* = 0.005). Meanwhile, a significant main effect for group was revealed for SHI-15′s speech function (*F*(1, 11) = 35.387, *p* < 0.001), total score (*F*(1, 11) = 29.691, *p* < 0.001), and psychosocial score (*F*(1, 11) = 22.214, *p* = 0.001), and SWAL-QOL’s communication (*F*(1, 11) = 17.035, *p* = 0.002) as shown in Table 3.

**Table 3:** Comparison of speech and swallowing-related quality of life in IPD and MSA-C groups before and after LSVT

Variables	IPD			MSA-C			Time		Group		Time*Group	
	Pretest	Posttest	Follow-up	Pretest	Posttest	Follow-up	*F*	*p*	*F*	*p*	*F*	*p*
SHI-15												
Speech function	6.50 ± 4.13	3.67 ± 5.08	8.17 ± 6.85	24.71 ± 4.53	21.57 ± 5.71	21.71 ± 9.89	1.319	.288	35.387	.000***	0.912	.416
Psychosocial	5.83 ± 5.98	2.50 ± 4.68*	3.17 ± 5.03*	22.29 ± 7.27	17.57 ± 6.32*	16.43 ± 9.03	4.156	.029*	22.214	.001**	0.464	.635
Total score	12.33 ± 9.13	6.17 ± 9.57	11.33 ± 10.89	47.00 ± 11.70	39.14 ± 11.68	38.14 ± 18.50	2.296	.124	29.691	.000***	0.757	.481
SWAL-QOL												
Burden	7.17 ± 2.48	8.33 ± 2.58	7.50 ± 2.42	6.43 ± 3.35	6.71 ± 3.45	7.29 ± 3.40	1.659	.213	0.283	.605	1.396	.269
Eating duration	7.33 ± 2.73	8.67 ± 2.65	8.83 ± 1.60	4.86 ± 3.48	6.71 ± 3.63	7.43 ± 2.69*	7.431	.003**	1.696	.219	0.465	.634
Eating desire	10.83 ± 1.60	12.33 ± 2.87	12.83 ± 2.99	10.29 ± 3.45	10.43 ± 4.75	10.86 ± 3.67	1.851	.181	0.722	.414	0.708	.504
Symptom frequency	59.33 ± 10.74	67.67 ± 1.63	64.00 ± 5.32	54.00 ± 12.76	60.43 ± 9.57*	58.14 ± 10.17	8.179	.002**	1.675	.222	0.144	.867
Food selection	9.17 ± 0.98	9.33 ± 1.63	9.33 ± 1.63	7.29 ± 2.87	7.57 ± 2.82	8.29 ± 2.36	1.460	.254	1.795	.207	0.857	.438
Communication	9.00 ± 0.89	9.50 ± 0.83	8.83 ± 0.98	4.43 ± 1.81	5.57 ± 2.57	5.00 ± 3.60	1.184	.325	17.035	.002**	0.260	.773
Fear	17.83 ± 3.92	19.17 ± 1.60	18.50 ± 3.20	15.43 ± 4.86	14.00 ± 6.63	16.29 ± 5.12	0.871	.432	1.781	.209	2.882	.077
Mental health	21.33 ± 5.31	23.17 ± 2.22	23.67 ± 2.06	18.43 ± 7.87	18.57 ± 8.50	17.43 ± 8.58	0.379	.689	1.797	.207	1.037	.371
Social functioning	22.17 ± 3.37	23.50 ± 3.20	22.00 ± 5.02	17.43 ± 8.73	16.57 ± 9.19	17.00 ± 9.22	0.091	.914	2.218	.165	0.450	.643
Sleep	7.50 ± 3.27	8.50 ± 3.50	9.00 ± 2.53	6.71 ± 2.28	8.14 ± 2.34	9.00 ± 4.04	2.260	.128	0.080	.783	0.095	.910
Fatigue	7.67 ± 3.07	10.00 ± 1.67	9.00 ± 1.54	7.57 ± 2.82	9.57 ± 3.78	7.14 ± 3.02	3.438	.050	0.437	.522	0.576	.570
Total score	179.33 ± 25.89	199.00 ± 16.39	193.50 ± 19.83	151.86 ± 46.14	164.86 ± 50.21*	163.86 ± 49.39	6.900	.005**	2.139	.172	0.267	.768

Data are presented as mean ± SD

*LSVT* Lee Silverman Voice Treatment, *IPD* Idiopathic Parkinson’s disease, *MSA-C* Multiple system atrophy-Cerebellar type, *MPT* Maximum phonation time, *SHI-15* Speech handicap index-short form, *SWAL-QOL* Swallowing-Quality of Life

*P < 0.05, **P < 0.01, ***P < 0.001

^*^significant results were returned for pretest and posttest, Pretest-Follow-up

### Changes in Speech and Swallowing-related Quality of Life in IPD and MSA-C Groups After LSVT

In the IPD group, a repeated-measure ANOVA revealed significant differences between pretest and posttest and between pretest and follow-up for SHI-15′s psychosocial score (*p* = 0.045; *p* = 0.025). In the MSA-C group, significant differences were revealed between pretest and posttest for SHI-15′s psychosocial score (*p* = 0.042) and SWAL-QOL’s symptom frequency (*p* = 0.037) and total score (*p* = 0.039). In addition, a significant difference between pretest and follow-up was revealed for SWAL-QOL’s eating duration (*p* = 0.012) as shown in Table 3.

## Discussion

Patients with PD may experience decreased maximal vocalization time due to reduced muscle strength in the respiratory-vocal system and decreased voice loudness due to the vocal cord muscle weakness [22]. In this study, the use of LSVT-LOUD achieved the goal of improving voice strength and vocalization ability. Following the voice therapy, MPT and voice intensity significantly increased in patients with PD, and these gains were sustained over 3-month follow-up period in the IPD group (Table 1).

In previous studies, MPT has been shown to be associated with oropharyngeal motor function, laryngeal elevation, and the triggering of pharyngeal swallowing [23, 24]. Patients with IPD and MSA-C experience pharyngeal impairment associated with incomplete upper esophageal sphincter relaxation, reduced upper esophageal sphincter opening, high intrabolus pressure, and abnormal pharyngeal wall motion [25]. Thus, the vocal folds in the laryngeal structure not only play a role in phonation but also act as a sphincter at the time of deglutition. The aryepiglottic folds in the larynx also serve as a guide through which food can pass laterally into the pyriform recess. Therefore, the larynx not only affects the ability to produce speech, but also facilitates bolus movement, protects the lower area of the airway, and prevents aspiration during swallowing [26]. In this study, we found significant post-treatment improvements in MPT and voice intensity, and the post-treatment scores for the pharyngeal phase with 15-ml thin liquid bolus swallows revealed significant improvement in both patient groups (Table 1). Additionally, both patient groups showed significant improvements after treatment in premature bolus loss (Table 2), which is evidence of improvement in oral phase motor function, as well. Therefore, increases in MPT and voice intensity were correlated with improvement of swallowing function in the oral and pharyngeal phases.

Premature bolus loss is a sign of reduced tongue strength and motility, as the tongue is the main structure that supports the bolus during transport into the pharynx [27]. In previous studies, LSVT has been shown to improve tongue strength and the oral phase of swallowing [12], consistent with the premature bolus loss improvements in this study. While the other sub-items in the oral phase did not show significant improvements, the baseline oral phase sub-item scores were all close to zero, with the exception of premature bolus loss in the MSA-C group. In addition, it has been noted that the pharyngeal phase showed significant improvements in both groups between pretest and posttest, but the only pharyngeal phase sub-item with significant improvement was laryngeal elevation in the IPD group. Based on the raw scores of the VDS, other sub-items such as pyriform sinus residue, coating on the pharyngeal wall, and pharyngeal transit time all show slight improvements between pretest and follow-up, but due to the small sample size, the results were not statistically significant. Further research with larger sample sizes may show more significant improvement in the VDS sub-scores.

Unlike patients with IPD, patients with MSA-C show severe dysarthria from the onset of illness [28, 29]. Thus, a significant group effect was found for speech parameters including MPT (Table 1) and quality of life (speech function, psychosocial function, total score of SHI-15; communication score of SWAL-QOL; Table 3). Nonetheless, in this study, patients with MSA-C showed significant improvements in measures related to speech function (MPT, voice intensity; Table 1) and quality of life (psychosocial function of SHI-15; Table 3) after treatment, and statistical analysis in comparison to IPD revealed a significant time effect, and no time × group interaction effect, across many measures (voice intensity, psychosocial function of SHI-15; Tables 1 and 3). This suggests that both patients with IPD and MSA-C experienced improvements in speech function and quality of life to a similar degree (Tables 1 and 3) 9.

In a previous study, intervention that trained the muscles involved in swallowing was found to improve the quality of life in patients with IPD for items related to symptom frequency on SWAL-QOL [30]. In the present study, the quality of life associated with swallowing improved slightly after LSVT-LOUD for both the IPD and MSA-C groups. When SWAL-QOL results were compared after treatment in IPD and MSA-C groups, respectively, the MSA-C patient group showed significant improvements in eating duration, symptom frequency, and total score (Table 3). Overall, the gains in speech function and speech-related quality of life in this study support the hypothesis that LSVT resulted in stronger respiration and laryngeal muscle movements, consequently improving the quality of life related to swallowing as reported by the IPD and MSA-C patients.

In patients with PD, swallowing problems occur as the disease progresses [31, 32]. Previous research suggests that LSVT-LOUD may improve pharyngoesophageal deglutitive function and cough reflex [11]. The results of this study showed that swallowing function scores on VDS were improved after LSVT (Tables 1 and 2). With these data, this study supported the hypothesis that LSVT enhances swallowing function especially in the pharyngeal phase. In addition, this intervention may be appropriate for patients with MSA-C who suffer from dysphagia.

NIH-SSS, with a maximum score of eight, quantifies signs of pharyngeal phase dysphagia, including vallecular residue, pyriform sinus residue, penetration, aspiration, as well as the clearance of the bolus through the upper esophageal sphincter. When we compared pre- and post-LSVT scores between the IPD and MSA-C groups, there was no significant difference in NIH-SSS score before and after treatment. However, there was a significant time effect for NIH-SSS score in patients with IPD and MSA-C (Table 1). It is important to note that the mean pre-treatment NIH-SSS score in both patient groups showed low severity, which can explain the lack of significant improvements post-treatment. Accordingly, this time effect supports the finding of improved swallowing function in the pharyngeal phase in both patient groups (Table 1).

Overall, the MSA-C group experienced slightly less improvement in speech and swallowing function compared to the IPD group, but both groups had a similar level of long-term maintenance of the improvement (Tables 1 and 2). The difference in the degree of improvement is likely because MSA-C typically shows a faster disease progression compared to IPD, and the MSA-C group began treatment at a higher average disease severity in terms of speech and quality of life function (Tables 1 and 3). Additionally, both groups showed limited improvements in swallowing function between pretest and follow-up, with significant improvements seen in the IPD group’s pharyngeal phase and premature bolus loss, and in none of the MSA-C group’s VDS scores (Tables 1 and 2). This is possibly a result of the degenerative nature of IPD and MSA-C [31, 32]. It is possible that MSA-C patients could benefit from periodic maintenance treatment sessions after completion of the standard LSVT-LOUD protocol. Further research with a longer follow-up period is necessary to investigate this phenomenon in more depth.

However, some limitations should be noted. First, we did not use a control group for this study. Second, the sample size in this study is small. This study should be considered as a pilot study to assess the clinical efficacy of LSVT as a treatment for swallowing problems. Further research investigating the effects on the swallowing function of LSVT in patients with IPD and MSA-C using a larger sample size and a control group is required.

## Conclusions

Previous studies have demonstrated the effect of LSVT on speech and swallowing function in IPD patients, and this study provided more evidence to this claim with significant improvements in speech and swallowing function and quality of life. Furthermore, this study showed that LSVT elicits significant improvements in MSA-C patients. We deemed LSVT to be an effective treatment for MSA-C because speech and swallowing functions measured by MPT, voice intensity, and VDS scores improved significantly after treatment, particularly in the pharyngeal phase. In addition, LSVT demonstrated a positive effect on speech- and swallowing-related quality of life, evidenced by the significant changes in SWAL-QOL and SHI-15 scores. We believe that the results of this study can contribute to the clinical foundation for LSVT in IPD and MSA-C patients with speech and swallowing problems.

## Supplementary Information

Below is the link to the electronic supplementary material.

Supplementary file1 (DOCX 28 KB)
