# Supplementary material for: Swallowing Outcomes Following Voice Therapy in Multiple System Atrophy with Dysphagia: Comparison of Treatment Efficacy with Parkinson’s Disease
Source: Dysphagia. 2021 Mar 5;37(1):198–206. doi: 10.1007/s00455-021-10265-9 (PMC8844176; doi:10.1007/s00455-021-10265-9)
Supplement: Supplementary file 1 — Supplementary file1 (DOCX 28 KB) [file 455_2021_10265_MOESM1_ESM.docx]

**Supplementary Results**

*Inter-rater reliability and intra-rater reliability for measuring NIH-SSS and VDS*

There was a significant correlation in the NIH-SSS scores with r = 0.812 ± 0.13 (range: 0.723–0.967) and VDS scores with r = 0.888 ± 0.06 (*P* = 0.01). The correlation of the NIH-SSS scale for randomly selected 20% data was r = 0.933 ± 0.06 (range: 0.863-0.986) and the VDS scale was r = 0.859 ± 0.82 (range: 0.767-0.924). The inter-rater reliability of NIH-SSS and VDS showed intraclass correlation coefficients (ICC) of 0.94 and 0.99, respectively, on 13 consecutive participants scored by three independent scorers. The intra-rater reliability for all subjects was determined for one investigator, and that of NIH-SSS and VDS showed ICCs of 0.89 and 0.99, respectively.
